# Pharmacological Action of *Mentha piperita* on Lipid Profile in Fructose-Fed Rats

**Published:** 2011

**Authors:** Rajendra Mani Badal, Divya Badal, Pourush Badal, Ashish Khare, Jyoti Shrivastava, Vinod Kumar

**Affiliations:** a Government Ayurvedic Hospital, Barua Sagar, Jhansi, U.P.-284001, India.; b Department of Biochemistry, Bundelkhand University, Jhansi, India.; c Department of Pharmacology, VNS Institute of Pharmacy, Neelbad, Bhopal-462044, MP, India.; d Smt. Vidyawati College of Pharmacy, Jhansi, U.P, India.; e Indian Drugs and Pharmaceuticals Limited, Rishikesh, U.T, India.

**Keywords:** Antioxidant defense, Fructose, Total cholesterol, Triglyceride hyperlipidemia, HDL, *Mentha piperita*.

## Abstract

Cardiovascular diseases with an incidence of approximately 50% are the main causes of death in most advanced countries and an increasing trend in the developing world as well. The World Health Organization estimates that 12 million people per year worldwide die from cardiovascular diseases. Cardiovascular diseases are becoming an increasing problem worldwide and hypercholesterolemia has been correlated for coronary heart diseases. Nearly all lipoproteins are involved in this process including cholesterol carried by very low density lipoproteins (VLDL), remnant lipoproteins and low density lipoproteins (LDL). Currently, available hypolipidemic drugs have been associated with the number of side effects. Herbal treatment for hyperlipidemia poses no side effects and is relatively cheap and locally available. In view of this, the present study was carried out to investigate the effect of Menthe piperita on serum lipid levels of albino rats. Mentha piperita aqueous extract (100 mg/Kg, 250 mg/Kg p.o. daily) was fed for 3 weeks on fructose-fed rats and the levels of glucose, cholesterol, triglycerides, very low density lipoprotein, low density lipoprotein, and atherogenic index was measured. Twenty-four male Sprague Dawley rats were divided into four groups (6 per group). The results of present study indicate that Mentha piperita had significant beneficial effects against fructose-induced hyperlipidemia and showed good antioxidant activity. The aqueous extract of the plant produced a significant decrease (p < 0.05) in elevated levels of glucose, cholesterol, triglycerides, very low density lipoprotein, low density lipoprotein and atherogenic index and also increased the high density lipoprotein cholesterol levels and HDL-ratio without affecting serum insulin levels in fructose-fed rats.

## Introduction

Increased fructose consumption in humans is believed to be one of the important factors for impaired glucose tolerance leading to diabetes (1). Epidemiologic and clinical evidences document a close association among hypertension, insulin resistance and dyslipidemia (hypertriglyceridemia and lower levels of high- density lipoprotein cholesterol) and they are also the predominant features of metabolic syndrome (2). Hyperinsulinemia**/**insulin resistance and hypertriglyceridemia have been documented in the fructose-induced hypertensive rat model (3). High-fructose-fed diet induces well characterized metabolic dysfunction, typically resulting in a rapid elevation of serum triglycerides with a corresponding increase in blood pressure in two weeks. Animals maintained on this diet for longer periods of time developed elevated free fatty acids and hyperinsulinemia at the expense of glycemic control. Fructose has also been shown to have pro-oxidant effects. Increased formations of lipid peroxidation end products and defects in free radical defense system have been documented in high fructose-fed rats (4). Thus, this animal model exhibits many of the hallmarks of an early stage of the Metabolic Syndrome (or “Syndrome X”). This cluster of changes appears to be the major risk factor in the pathogenesis of coronary artery disease. *Mentha piperita *is a popular worldwide herbal tonic. It is a perennial, glabrous, strong scented herb from the family Menispermaceae and generally used as a flavoring agent. It has been a popular home remedy for digestive ailments for two centuries in India. The volatile oil obtained from this plant, known as mint or peppermint oil, is used as antiseptic, stimulant, carminative and for allaying nausea and vomiting and also has got commercial value. The major components of this oil are menthofuran, menthol, menthyl acetate, neomenthol, menthone and isomenthone. The plant has been used for antinociceptive, anti-inflammatory, antimicrobial and antioxidant properties. The flavonoids namely eriocitrin, narirutin, hesperidin, luteolin-7-O-rutinoside, isorhoifolin, diosmin, rosmarinic acid, and 5, 7-dihydroxycromone-7-O-rutinoside isolated from the plant showed antiallergic effects. Menthone is also a major constituent of the plant (5). The essential oil has also both antibacterial and antifungal properties. The major constituents of the essential oil are: menthol, menthone, pulegone, menthofuran, menthyl acetate and isomenthone. The leaves contain flavonoid glycosides, eriocitrin, luteolin-O-rutinoside, hesperidin, isorhoifolin, azulenes, cholene and carotenes. Peppermint oil relaxes carbachol contracted guinea-pig tenia coli and inhibits spontaneous activity in guinea-pig colon and rabbit jejunum. It relaxes gastrointestinal smooth muscle by reducing calcium influx. Peppermint oil reduces gastric emptying time in dyspeptics. The aqueous and ethanol extracts exhibited antiviral activity against RPV (rinderpest virus), a highly contagious viral disease of cattle (6).

## Experimental

*Plant material*

The plant material used in this study was collected in January from region of Madhya Pradesh. The plant was identified and authenticated by Dr. Zia ul Hassan, professor of department of botany Safia, P.G. Science College, Bhopal. Voucher specimen No. 128/botany/Safia/2010, India.

*Extraction*

*Preparation of plant extract*

Dry menthe leaves (51.5 g) were extracted by adding 500 mL of distilled water and boiling for 30 min. Then the extract was filtered and the filterate evaporated to dryness using evaporator. This extract was mixed with 5% CMC and used for various experimental purposes.

*Experimental animals and treatment protocol*

Male Sprague Dawley rats weighing 200-250 g housed under well controlled conditions of temperature (22 ± 2°C) and humidity (55 ± 5%) and also in 12/12 h (light/dark) cycle were given access to food and water *ad libitum*.

The protocol of the experiment was approved by the Institutional Animal Ethical Committee as per the guidance of the Committee for the Purpose of Control and Supervision of Experiments on Animals (CPCSEA), the Ministry of Social Justice and Empowerment, Government of India.

Twenty-four male Sprague Dawley rats were divided into four groups of six:

Group 1: Normal diet and water ad libitum and received vehicle solvent. Group 2: Normal diet and water with 10% fructose and received vehicle solvent (Control). Group 3: Normal diet and water with 10% fructose and received aqueous extract of plant (100 mg/Kg).Group 4: Normal diet and water with 10% fructose and received aqueous extract of plant (250 mg/Kg).

Each group was administered vehicle or drugs daily for 3 weeks through gavage using oral feeding needle. Feeding of animals by fructose and extract were done simultaneously from the beginning. At the end of 3 weeks period, animals were kept fasting overnight and the blood samples were collected from the retro orbital plexus in the centrifuge tube. The blood samples were allowed to clot for 30 min at room temperature and then centrifuged at 5000 rpm for 15 min. Serum samples thus obtained were stored at -4°C until biochemical estimation was carried out. After blood collection, animals were killed using deep ether anesthesia and the liver was excised and subjected to the antioxidant estimation.

*Biochemical analysis*

The serum parameters were analyzed spectrophotometrically by using double beam UV-Visible spectrophotometer (Shimadzu UV-Visible spectrophotometer, model 1700). The estimation of serum glucose (GOD-POD method), cholesterol (enzymatic method), triglyceride (enzymatic method) and HDL-cholesterol (phosphotungstate method) was carried out using respective diagnostic kits (Bayer Diagnostic Ltd., India). Serum insulin was estimated by a radioimmunoassay method from Bhabha Atomic Research Center, Mumbai, India. In addition to the above parameters, body weight, food intake and water intake of animals were recorded. VLDL-cholesterol and LDL-cholesterol were calculated as per Friedewald›s equation (7).

VLDL = Serum triglyceride/5

LDL = Total serum Cholesterol - Total serum triglycerides / 5 - Total serum HDL-C

HDL ratio = HDL–Cholesterol × 100 / Total serum Cholesterol - HDL-C

Atherogenic Index (8) = Total serum triglycerides / Total serum HDL-C

*Estimation of antioxidants*

After 3 weeks, animals were sacrificed; the liver was quickly removed and washed in ice-cold saline. One hundred mg of liver tissue was homogenized in ice-cold trihydrochloride buffer (pH = 7.2). The homogenate was centrifuged at 800 rpm for 10 min, followed by centrifugation of the supernatant at 12,000 rpm for 15 min. The obtained supernatant was used for the estimation of reactive oxygen metabolites in terms of lipid peroxidation (9), superoxide dismutase (SOD) (10), catalase (11), reduced glutathione (GSH) (12) and total protein estimation (13).

*Statistical analysis*

Results were analyzed statistically using one way analysis of variance (ANOVA) followed by Dunnett’s test. Data were considered statistically significant at p < 0.05.

## Results and Discussion

*Effects on body weight, glucose, insulin and lipid profile*.

Fructose-fed rats exhibited significant increase in body weight as compared to normal control rats (p < 0.05). Treatment with extracts in fructose-fed rats reversed this increase in body weight (p < 0.05). Fructose-fed rats were hyperglycemic and hyperinsulinemic as compared to normal control animals (p < 0.05). Treatment with extract in fructose-fed rats reduced glucose level without affecting insulin levels (p < 0.05) (Table1). Fructose-fed animals exhibited significantly higher serum cholesterol, triglyceride, VLDL-cholesterol and LDL-cholesterol levels whereas there was a decrease in HDL-cholesterol and HDL-ratio as compared to normal control animals. Extract treatment in fructose-fed rats produced a significant decrease in serum cholesterol, triglycerides, VLDL-cholesterol and LDL-cholesterol levels, with an increase in HDL-cholesterol and HDL-ratio (Table 1). Furthermore, extract treatment to fructose-fed rats exhibited significant improvement in atherogenic index (Table 1). 

**Table 1:** Effects of Treatment of *Mentha piperita *leaves extract on general characteristics of the fructose induced hyperlipidemia and hyperinsulinemia

**Group**	**Normal**	**Fructose-fed control**	**Fructose-fed treated with extract** **(100 mg/Kg)**	**Fructose-fed treated with extract** **(250 mg/Kg)**
**Characteristic**				
**Body weight (g/rat)**	228 ± 9.564	334 ± 12.453	292 ± 13.483	273 ± 8.561**
**Water intake (mL/rat/day)**	43 ± 7.325	46 ± 5.687	41 ± 7.230	47 ± 4.624
**Food intake (g/rat/day)**	15.8 ± 2.781	18.7 ± 1.569	15.9 ± 1.989	16.5 ± 1.349
**Serum Glucose (mg/dL) **	75.4 ± 7.620	117.3 ± 4.945*	86.0 ± 5.060*	76.4 ± 6.514**
**Serum Insulin (μU/mL)**	17.1 ± 4.385	42.5 ± 4.660*	35.6 ± 6.898	31.8 ± 2.358
**Serum Cholesterol (mg/dL)**	83.1 ± 7.556	135 9.125*	103.5 ± 11.105	94.33 ± 6.647**
**Serum HDL (mg/dL)**	33.8 ± 2.548	14.7 ± 2.088*	22.6 ± 2.765**	29.8 ± 1.797**
**Serum Triglyceride (mg/dL)**	99.1 ± 11.649	166.8 ± 8.920*	99.8 ± 8.495**	90.8 ± 9.793**
**SerumVLDL (mg/dL)**	19.8 ± 2.330	33.3 ± 1.784*	19.9 ± 1.699**	18.1 ± 1.959**
**Serum LDL (mg/dL)**	42.6 ± 8.710	87.4 ± 10.538*	53.7 ± 11.858**	40.5 ± 15.157
**HDL Ratio**	37.7 ± 3.903	12.2 ± 2.504*	45.1 ± 7.622**	58.8 ± 3.956**
**Atherogenic index:**	4.7 ± 0.878	11.5 ± 1.112*	3.4 ± 0.438**	2.7 ± 0.369**

n = 6; *: Significantly different from normal control, p < 0.05; **: Significantly different from fructose-fed control, p < 0.05.

*Effects on antioxidant defenses*

Fructose-fed animals showed significant increase in lipid peroxidation in terms of malondialdehyde amount and superoxide dismutase (SOD) in liver tissue homogenates when compared to normal control animals. Treatment with extracts in fructose-fed rats significantly decreased lipid peroxidation and increased SOD in liver tissue homogenates (p < 0.05) (Table 2). Fructose-fed rats showed significant decrease in catalase and glutathione levels in liver tissue homogenate in comparison with normal control animals. Treatment with extract significantly increased catalase and glutathione levels in liver tissue homogenate (p < 0.05) (Table 2).

**Table 2:** Effects of Treatment of *Mentha piperita *fruit extract on antioxidant parameters in fructose-induced hyperlipidemia and hyperinsulinemia

**Group**	**Normal**	**Fructose-fed control**	**Fructose-fed treated with extract ** **(100 mg/Kg)**	**Fructose-fed treated with extract ** **(250 mg/Kg)**
**Parameter**				
**Lipid peroxidation (μg/mg)**	1.1 ± 0.101	4.9 ± 0.340*	3.1 ± 0.244**	1.8 ± 0.280**
**Superoxide dismutase ** **(U/mg of protein)**	0.22 ± 0.0850	0.08 ± 0.011*	0.175 ± 0.010**	0.20 ± 0.009**
**Catalyst (U/mg of protein)**	24.34 ± 1.847	12.46 ± 3.100*	17.77 ± 1.983**	29.25 ± 1.440**
**Glutathione (μmole/mg of protein)**	5.047 ± 0.680	1.952 ± 0.539*	2.847 ± 0.365**	4.485 ± 0.700**

n = 6; *: Significantly different from normal control, p < 0.05; **: Significantly different from Fructose-fed control, p < 0.05

The prevalence of insulin resistance and associated diseases has risen seriously around the world. The general view of insulin action places this hormone at the point of multiple organ adaptations to the ingested nutrients, in particular, dietary carbohydrates. It has been established that insulin resistance, impaired glucose tolerance, hyperinsulinemia, hypertension and hyperlipidemia are associated with fructose intake in animal models. Increasing the dietary fructose consumption might be one of the factors responsible for the development of obesity and the accompanying insulin resistance syndrome (14). Thus, the rats received 3 weeks of fructose-rich water could be served as a reliable model for the investigation of insulin resistance (15).

In the present study, an oral administration of aqueous leaves’ extract of *Mentha piperita *was found to decrease the plasma glucose concentration of fructose-fed rats in a dose-dependent manner, showing the beneficial action of *Mentha piperita *in rats with insulin resistance. 

The mechanism of glucose lowering action might involve proceedings other than pancreatic *β *cells insulin secretion since we did not observe any improvement in insulin resistance in our study. Fructose-fed rats exhibited clear cut abnormalities in lipid metabolism as a proof for a significant elevation of plasma total cholesterol, triglycerides, LDL-C, atherogenic index and reduction of HDL-C levels. Treatment with aqueous extract of *Mentha piperita *leaves’ extract for 21 days significantly reduced serum total cholesterol, triglycerides and LDL-C associated with concomitant significant increase in HDL-C levels and decrease in atherogenic index in hyperlipidemic rats indicating its potent antihyperlipidemic and antiatherogenic activity. The glucose lowering action of extract can be due to the improved lipid metabolism apart from the direct interaction with glucose homeostasis. The triglyceride lowering properties (activity) of extract can indirectly contribute to the overall antihyperglycemic activity through a mechanism called glucose-fatty acid cycle (16, 17). According to the Randle’s glucose-fatty acid cycle, increased supply of plasma triglycerides per se can constitute a source of increased free fatty acid (FFA) availability and oxidation that can impair insulin action, glucose metabolism and utilization leading to development of hyperglycemia. It has been postulated that fructose can accelerate free radical production similar to glucose. For example, Suzuki K (18) has observed an increased production of H_2_O_2 _and formation of hydroxyl radicals by hamster pancreatic cells incubated with fructose in the presence of a metal ion catalyst. Furthermore, due to hyperglycemia, an increase in non-enzymatic glycosylation occurs, accompanied with glucose oxidation and these reactions are catalyzed by Cu^+2^ and Fe^+2^, resulting in formation of O_2_ - and OH radicals which further accelerates the risk of cardiac diseases in dyslipidemia (19). Lipid peroxidation is one of the characteristic features of chronic fructose consumption. In this context, a marked increase in the concentration of TBARS was observed in liver of fructose-fed rats. Increased lipid peroxide concentration in the liver of fructose-fed animals has already been reported (20). Administration of the extract significantly decreased the levels of TBARS in fructose-fed rats (Table 2). Glutathione (GSH), a tripeptide present in all the cells is an important antioxidant. Decreased glutathione levels in fructose-fed animals have been considered to be an indicator of increased oxidative stress. GSH also acts as a free radical scavenger in the repair of radicals caused biological damage. A decrease was observed in liver GSH in fructose-fed animals. Administration of the extract increased the content of GSH in liver of fructose-fed rats (Table 2). The cellular radical scavenging systems include the enzymes such as superoxide dismutase (SOD), which scavenges the superoxide ions by catalyzing its dismutation and catalase (CAT), a heme enzyme which removes hydrogen peroxide. Therefore, reduction in the activity of these enzymes (SOD, CAT) results in a number of deleterious effects due to the accumulation of superoxide anion radicals and hydrogen peroxide. Administration of aqueous extract increased the activity of SOD and catalase in fructose-fed rats. Since the extract showed *in-vivo *antioxidant activity in fructose-fed rats, improvement of the liver functions and the subsequent increase in the uptake and utilization of blood glucose might be the mechanism of action of this extract as glucose lowering and hypolipidemic agent.
